# Political Affiliation, Policy Measures, and Intention to Receive COVID-19 and Influenza Vaccines

**DOI:** 10.3390/ijerph21121608

**Published:** 2024-11-30

**Authors:** Isabel J. Ricke, Alicen B. Spaulding, Nickolas N. Rajtar, Lauren Lundberg, Ruby H. N. Nguyen

**Affiliations:** 1 Division of Epidemiology & Community Health, School of Public Health, University of Minnesota, Minneapolis, MN 55454, USA; 2 Vaccine Research Center, National Institute of Allergy and Infectious Diseases, National Institutes of Health, Bethesda, MD 20892, USA; 3 Department of Plant Pathology, University of Minnesota, St. Paul, MN 55108, USA

**Keywords:** COVID-19, influenza, vaccines, vaccine mandates, politics

## Abstract

Our study aimed to assess the impact of political affiliation, personal beliefs, and policy measures on the intention to receive routine COVID-19 and influenza vaccines in the coming year. A cross-sectional study of 1256 individuals at Minnesota State and County Fairs was conducted to assess their intention to receive COVID-19 booster and influenza vaccines in the coming year. The association between vaccine intention and political affiliation, belief in collective responsibility, and workplace/school vaccine requirements were analyzed using multinomial logistic regression. Vaccine intention in the coming year was high among our participants; 65% intended to receive both vaccines, 11% intended to receive only the influenza vaccine, 7% intended to receive only the COVID-19 vaccine, and 17% planned to receive neither. Political affiliation was strongly associated with the intention to receive both vaccines. Republicans were far more likely than Democrats to report plans to receive neither vaccine (aOR: 12.8; 95% CI: 6.2–26.6), or only the influenza vaccine in the coming year (aOR: 8.7; 95% CI: 4.2–17.9). Additionally, those who planned to receive both vaccines were significantly more likely to view vaccines as a collective responsibility. This study highlights the significant influence of political affiliation and beliefs in collective responsibility on vaccine intentions.

## 1. Introduction

The emergence of the COVID-19 pandemic necessitated a global health response unprecedented in recent history, with the development and distribution of vaccines at the forefront of the efforts to control the spread of the virus [1,2,3,4]. A year after the first identification of COVID-19, the United States (US) Food and Drug Administration (FDA) granted Emergency Use Authorization to Pfizer-BioNTech, Moderna, and J&J/Janssen to produce and manufacture COVID-19 vaccines [5]. By August 2021 and January 2022, the Pfizer-BioNTech and Moderna COVID-19 vaccines, respectively, had received full FDA approval [6,7]. In September 2022, updated bivalent vaccines from both manufacturers were endorsed by the Centers for Disease Control and Prevention (CDC). For both the 2023–2024 and 2024–2025 seasons, updated COVID-19 vaccines from Pfizer-BioNTech, Moderna, and Novavax were available and recommended for everyone aged 6 months and older [8]. While the future of COVID-19 vaccine recommendations in the US remains somewhat uncertain, there are indications that suggest that the vaccine will be updated and recommended annually, mirroring the approach taken with the seasonal influenza vaccine [9]. With the pending potential for the co-administration of COVID-19 and influenza vaccines, it will be important to distinguish what factors influence the acceptance of these vaccines to identify targets to improve vaccination rates for both viruses. 

Policy measures such as workplace vaccine mandates have been used to attempt to increase the acceptance of COVID-19 and influenza vaccines. There is a precedent for vaccine mandates in the US. One of the earliest examples is the 1904 Supreme Court case *Jacobson v Massachusetts*, which upheld a mandate requiring the smallpox vaccine for adults in Massachusetts, affirming the authority of states to enforce compulsory vaccination for public health [10]. This ruling laid the legal groundwork for vaccination mandates in the US. During the COVID-19 pandemic, mandates were implemented for specific populations to control the spread of the virus. These mandates targeted federal contractors, healthcare workers, private sector employees, federal government employees, and military personnel [11]. While influenza vaccinations are not mandated in Minnesota, many employers, mainly healthcare systems, have enacted workplace policies requiring influenza vaccination [12,13,14]. Understanding the impact of workplace vaccination requirement policies on an individual’s intention to receive vaccines, especially when considered alongside other key factors shaping vaccine attitudes and behaviors, is crucial. 

The intersection of health behavior and political affiliation has emerged as a potent force shaping vaccine attitudes and behaviors [15]. Studies have documented a clear divide among political groups in COVID-19 vaccine acceptance rates [16,17,18]. Pew Research Center data revealed that while Republicans had a 15 percentage-point lower COVID-19 vaccine uptake compared to Democrats when the vaccine first became available in 2021, this gap increased to a 42% difference for the 2023–2024 updated COVID-19 vaccine [19]. Other studies have highlighted substantial differences in excess mortality rates along political lines, with registered Republicans experiencing a 10.4 percentage-point higher excess death rate compared to Democrats since COVID-19 vaccines became widely available [20]. This divide is likely due, in part, to differences in COVID-19 vaccine uptake across political groups, especially in regions with lower vaccination rates where excess mortality among Republicans has been markedly higher [20]. The concept of collective responsibility, emphasizing the importance of vaccines for community protection and reducing infectious disease transmission, may be critical in bridging ideological divides in vaccination behavior [21]. 

There remains a knowledge gap in how political affiliation can influence ongoing willingness to receive routine COVID-19 vaccines and other recommended vaccinations, such as the seasonal influenza vaccine. Specifically, it is unclear how deep the political divide runs in the context of newer vaccine doses and whether changing dynamics and public health messaging after the end of the public health emergency might have altered previous patterns of vaccine acceptance. The primary objective of this study was to assess the impact of political affiliation, policy measures, and personal beliefs on the intention to receive routine COVID-19 and influenza vaccines. We assessed these factors in a population-based cross-sectional study of participants in a liberal-leaning US state. 

## 2. Materials and Methods

This study was a cross-sectional survey conducted at the 2023 Minnesota State Fair and two Minnesota County Fairs (Stevens and Beltrami), all held between August and September 2023 [22]. Individuals were eligible for the study if they were 18 years or older and could read English. Eligible participants completed a self-administered online survey on-site via a tablet [23]. The University of Minnesota Institutional Review Board provided approval for this study.

Overall, 1256 participants enrolled in the study. Thirty participants did not complete the survey questions related to the primary outcome. Another 351 participants did not complete at least one of the questions related to a key predictor or relevant demographics. This resulted in a final analytic dataset consisting of 867 participants. 

Our primary outcome was the participant’s combined intention to receive both a recommended COVID-19 and routine influenza vaccine in the coming year. Participants were asked how likely they were to receive the COVID-19 or influenza vaccine this year, either together or separately. These questions were adapted from the CDC’s Vaccine Confidence Survey Question Bank [24]. Responses to both questions were on a 5-point Likert scale from “Extremely unlikely” to “Extremely likely”. Participants who indicated they were “Likely” or “Extremely likely” to accept the vaccine were categorized as intending to vaccinate.

Additionally, participants were asked about their previous COVID-19 vaccination status with a series of three questions: “Have you received a COVID-19 vaccine?”, “How many COVID-19 vaccine doses have you received (including primary series and booster vaccines)?”, and “Have you received a COVID-19 booster vaccine dose since September of 2022 (otherwise known as an updated or bivalent booster)?”. These questions were also adapted from the CDC’s Vaccine Confidence Survey Question Bank [24]. If participants indicated that they had not received a COVID-19 vaccine or they had not received a bivalent booster vaccine, they were asked for the primary reason why they had not. Similarly, participants were asked if they had ever received an influenza vaccine and if they had received an influenza vaccine in the past year. For those who had not received an influenza vaccine, either ever or within the past year, a follow-up question was included to identify their primary reason for not receiving it.

Key predictors for this study included political affiliation, COVID-19 and influenza workplace vaccination requirements, and belief in vaccines as a collective action. Participants self-reported their political affiliation as “Democrat”, “Republican”, “Independent”, or “Other”. Belief in vaccines as a collective responsibility was assessed through a validated measure from the 5C scale of psychological antecedents of vaccination, “Vaccination is a collective action to prevent the spread of diseases” [25]. The response options were on a 5-point Likert-style scale from “Strongly disagree” to “Strongly agree”. Finally, participants were asked about workplace vaccine requirements. Participants were asked whether they were required to receive the COVID-19 vaccine currently, previously, or not at all. They were also asked if their workplace currently required them to receive an influenza vaccine.

The primary outcome of our analysis was the combined intention to receive an updated COVID-19 vaccine and/or influenza vaccine in the coming year if it was recommended. We tabulated counts and proportions of study participants for each demographic and health characteristic stratified by their combined vaccine intention. We used bivariate statistical tests, including the Kruskal–Wallis rank sum test for continuous variables, Fisher’s exact test for categorical variables, and Pearson’s chi-squared test for binary variables. 

We used multinomial logistic regression to assess the impact of political affiliation, belief in vaccines as a collective responsibility, and vaccine requirements on the intention to receive COVID-19 and influenza vaccines in the coming year. Our dependent variable was categorical vaccine intention, which was categorized as the intention to receive only the influenza vaccine, only the COVID-19 vaccine, or both the COVID-19 and influenza vaccines. The intention to receive both category was the reference category. This model also adjusted for potential confounders identified via a conceptual model a priori. These covariates included age, gender, annual household income, educational attainment, and self-reported medical conditions that would increase the risk of severe illness from COVID-19. R version 4.1.3 was used for all analyses [26].

## 3. Results

The demographics of the 867 participants included in the analytic dataset are described in Table 1. The average age of participants was 48 (SD: 18), and 62% of participants were female. The majority of participants were also white (87%) and non-Hispanic (97%). Additionally, most participants had at least a college degree (70%) and a household income of at least USD 50,000 per year (83%). Most participants were Democrats (63%), while only 15% reported Republican affiliation, 15% reported being Independent, and 7% reported other political affiliation. Compared to the overall political makeup of Minnesota, where 46% identify as Democrats, 39% as Republicans, and 15% have no political leaning, our study population has a higher proportion of Democrats and a lower proportion of Republicans [27]. 

Table 2 describes the primary reason participants reported for declining each vaccine type in the past. For those who had never received a COVID-19 vaccine, concerns about side effects (35%) and doubts about vaccine effectiveness (26%) were the most frequently cited reasons. In contrast, those who chose not to receive the COVID-19 bivalent booster were most likely to state that they were not worried about getting sick (21%) or cited “other” reasons (27%). Respondents who had never been vaccinated for influenza primarily reported being unconcerned about getting sick (27%) or questioned the vaccine’s effectiveness (25%). Those who had not received an influenza vaccine in the past year also primarily reported a lack of concern about getting sick (26%) or cited “other” reasons (26%). 

Table 3 shows the results of the adjusted multinomial logistic regression modeling the combined effect of political affiliation, belief in vaccines as a collective responsibility, and COVID-19 and influenza workplace vaccination requirements. Compared to Democrats, Republicans had higher odds of not planning to receive either the COVID-19 vaccine or the influenza vaccine in the coming year (aOR: 12.8; 95% CI: 6.2–26.6). This was also observed in the Independents (aOR: 4.3; 95% CI: 2.1–8.7) and the “other” political affiliation group (aOR: 3.2; 95% CI: 1.3–7.6). 

Similarly, political affiliation was a strong predictor of plans to receive only the influenza vaccine as opposed to both influenza and the COVID-19 vaccine in the coming year. Compared to Democrats, Republicans had the highest odds of planning to receive only the influenza vaccine rather than both vaccines (aOR: 8.7; 95% CI: 4.2–17.9), followed by Independents (aOR: 3.2; 95% CI: 1.6–6.4), and those with “other” political affiliation (aOR: 3.0, 95% CI: 1.2–7.2). 

Belief in vaccines as a collective action was also strongly associated with combined vaccine intention. The adjusted odds of strongly believing in vaccination as a collective action were 94% lower (95% CI: 89–97% lower) in those who did not plan to receive either vaccine. 

Workplace vaccine requirements were also associated with intention, but these differences did not reach statistical significance for all measures. Those who intended only to receive the COVID-19 vaccine in the coming year had higher odds of having a workplace COVID-19 vaccine requirement (aOR: 2.0; 95% CI: 0.94–4.4) and much lower odds of having a workplace influenza vaccine requirement (aOR: 0.16; 95% CI: 0.05–0.6) compared to people who worked at workplaces without mandates. Results were similar for those working at a workplace with an influenza vaccine requirement. 

## 4. Discussion

This study highlights significant determinants of vaccine intentions for COVID-19 and influenza, emphasizing the role of political affiliation, beliefs about collective responsibility, and workplace vaccination requirements. Our results indicate that political affiliation is highly associated with yearly vaccine acceptance. In our study, individuals identifying as Republicans and Independents were significantly less likely than Democrats to intend to receive either vaccine. These findings align with previous research highlighting the substantial political divides in COVID-19 vaccination behaviors [20,28,29]. Conservative media exposure has been shown to be associated with lower COVID-19 vaccine intentions [29]. Additionally, recent studies show that counties with higher concentrations of Republican voters not only have lower vaccination rates but also higher numbers of COVID-19 cases and deaths, underscoring the health impact of political divides in vaccine uptake [20,28]. This evidence demonstrates the substantial influence of political affiliation on vaccine acceptance. It highlights the importance of targeted public health strategies to bridge ideological divides to reduce vaccine hesitancy and its wider health consequences. 

Our analysis also suggests that viewing vaccination as a collective responsibility is associated with higher odds of COVID-19 and influenza vaccine intention. This is consistent with the existing literature that has shown a positive association between collective responsibility and other prosocial beliefs and vaccine uptake [21,30,31,32]. Collective responsibility is fundamental to the success of public health, particularly as it relates to vaccines, which serve to protect both individuals and the communities they belong to. Framing vaccination as a shared societal obligation may help bridge divides and foster greater public acceptance of vaccination as an essential public health measure [33]. 

While not uniformly statistically significant, those with a workplace vaccination requirement were more likely to accept the vaccine associated with that requirement and less likely to receive the non-required vaccine. Vaccine mandates in US workplaces varied widely, and we do not know the details about the specific types of requirements our participants were subject to. This variability may have influenced participants’ responses and could have impacted our statistical analysis. Prior research about the effectiveness of vaccine mandates has been mixed. A multinational study found that government-mandated proof-of-vaccination requirements had a sizable and statistically significant impact on COVID-19 vaccine uptake [34]. On the other hand, an analysis of US state-level vaccine mandates and population-level vaccination suggested that COVID-19 vaccine mandates may be ineffective and may reduce the adoption of other voluntary vaccines [35]. Despite mixed evidence, mandates remain an essential component of public health strategy, particularly in healthcare settings and schools, and especially if they are perceived as a measure to increase collective protection [33]. Enhancing the public understanding of mandates as safeguards for community health may further mitigate resistance to vaccination requirements and increase vaccine uptake. 

This study has a few limitations worth noting. We drew from a convenience sample, which was not fully representative of the general population but did approximate the demographics of State Fair attendees [36]. Additionally, those distrustful of scientific studies or vaccination may have chosen not to participate, and individuals most concerned about COVID-19 may not have attended a crowded event such as the State Fair. Furthermore, 81 participants did not report their political affiliation. However, we performed a sensitivity analysis that included these additional 81 individuals and found little variation to our estimates and no difference in our interpretation of results. Additionally, 351 participants were excluded from our analysis due to missing data on at least one key predictor or another covariate. To assess potential non-response bias, we also reviewed the characteristics of these participants and found that they were similar in age, race, and socioeconomic status to those in the analytic dataset. This suggests that there were minimal differences between excluded and included participants. 

## 5. Conclusions

Our study underscores the complex interplay of political affiliation and policy measures in shaping vaccine intentions, highlighting how ideological beliefs can impact public health efforts. This relationship emphasizes the importance of public health messaging that frames vaccination as a community-oriented action essential for protecting vulnerable populations from infectious diseases. To bridge ideological divides and increase vaccine uptake, public health strategies should focus on fostering a sense of collective responsibility and reinforcing the benefits of vaccination for both individual and community well-being. 

## Figures and Tables

**Table 1:** Characteristics of survey respondents from convenience sample at Minnesota State and County Fairs (August 2023) by intention to receive routine COVID-19 and influenza vaccines in the coming year (N = 867).

Characteristic	Overall	Both COVID-19 and Influenza Vaccines	COVID-19 Vaccine Only	Influenza Vaccine Only	Neither COVID-19 Nor Influenza Vaccine	*p*-Value
	N = 867	N = 593	N = 59	N = 88	N = 127	
Age						<0.001
Mean (SD)	48 (18)	50 (18)	45 (18)	43 (17)	41 (17)	
Gender						0.009
Female	536 (61.8%)	386 (65.1%)	37 (62.7%)	50 (56.8%)	63 (50%)	
Male	331 (38.2%)	207 (34.9%)	22 (37.3%)	38 (43.2%)	64 (50%)	
Race						
White	743 (86.9%)	519 (88.5%)	46 (79%)	75 (87.2%)	103 (82.4%)	
American Indian or Alaskan Native	12 (1.4%)	5 (0.9%)	3 (5.2%)	2 (2.3%)	2 (1.6%)	
Black or African American	17 (2.0%)	10 (1.7%)	2 (3.4%)	2 (2.3%)	3 (2.4%)	
Asian	40 (4.7%)	25 (4.3%)	6 (10%)	1 (1.2%)	8 (6.4%)	
Other	19 (2.2%)	9 (1.5%)	1 (1.7%)	4 (4.7%)	5 (4.0%)	
Multiple Races	24 (2.8%)	18 (3.1%)	0 (0%)	2 (2.3%)	4 (3.2%)	
Unknown	12	7	1	2	2	
Hispanic ethnicity						0.9
Hispanic or Latino	28 (3.4%)	19 (3.3%)	1 (1.9%)	3 (3.5%)	5 (4.3%)	
Not Hispanic or Latino	806 (96.6%)	562 (96.7%)	51 (98.1%)	82 (96.5%)	111 (95.7%)	
Unknown	33	12	7	3	11	
Education						
Less than high school	0 (0%)	0 (0%)	0 (0%)	0 (0%)	0 (0%)	
High school graduate or equivalent (e.g., GED)	59 (6.8%)	30 (5.1%)	5 (8.5%)	10 (11.4%)	14 (11.0%)	
Some college, including associate degree or trade school	201 (23.2%)	120 (20.2%)	13 (22.0%)	20 (22.7%)	48 (37.8%)	
Bachelor’s degree	291 (33.6%)	184 (31.0%)	25 (42.4%)	32 (36.4%)	50 (39.4%)	
Graduate degree	316 (36.4%)	259 (43.7%)	16 (27.1%)	26 (29.5%)	15 (11.8%)	
Annual household income						
Less than USD 20,000	45 (5.2%)	25 (4.2%)	5 (8.5%)	9 (10.2%)	6 (4.7%)	
USD 20,000–49,999	107 (12.3%)	54 (9.1%)	15 (25.4%)	12 (13.6%)	26 (20.5%)	
USD 50,000–99,999	300 (34.6%)	205 (34.6%)	20 (33.8%)	30 (34.1%)	45 (35.4%)	
Over USD 100,000	415 (47.9%)	309 (52.1%)	19 (32.2%)	37 (42.0%)	50 (39.4%)	
Medical condition						0.001
Yes	186 (21.5%)	149 (25.1%)	9 (15.3%)	12 (13.6%)	16 (12.5%)	
No	681 (78.5%)	444 (74.9%)	50 (84.7%)	76 (86.4%)	111 (87.4%)	
Political affiliation						
Democrat	544 (62.7%)	451 (76.1%)	43 (72.8%)	27 (30.7%)	23 (18.1%)	
Republican	128 (14.8%)	34 (5.7%)	6 (10.2%)	30 (34.1%)	58 (45.6%)	
Independent	133 (15.3%)	80 (13%)	7 (11.9%)	19 (21.6%)	27 (21.3%)	
Other	62 (7.2%)	28 (4.7%)	3 (5.1%)	12 (13.6%)	19 (15.0%)	
Belief in vaccines as collective action						
Strongly agree	691 (79.7%)	554 (93.4%)	52 (88.1%)	44 (50.0%)	41 (32.3%)	
Moderately agree	112 (12.9%)	30 (5.1%)	4 (6.8%)	36 (40.9%)	42 (33.1%)	
Neutral	30 (3.5%)	3 (0.5%)	3 (5.1%)	5 (5.7%)	19 (15.0%)	
Moderately disagree	16 (1.8%)	3 (0.5%)	0 (0%)	1 (1.1%)	12 (9.4%)	
Strongly disagree	18 (2.1%)	3 (0.5%)	0 (0%)	2 (2.3%)	13 (10.2%)	
Current COVID-19 vaccine requirement						0.021
Yes	127 (14.6%)	91 (15.3%)	12 (20.3%)	16 (18.2%)	8 (6.3%)	
No	740 (85.4%)	502 (84.7%)	47 (79.7%)	72 (81.8%)	119 (93.7%)	
Current influenza vaccine requirement						<0.001
Yes	121 (13.9%)	91 (15.3%)	3 (5.1%)	23 (26.1%)	4 (3.1%)	
No	746 (86.1%)	502 (84.7%)	56 (94.9%)	65 (73.9%)	123 (96.9%)	

**Table 2:** Primary reason for declining COVID-19 primary series, COVID-19 booster vaccine, any influenza vaccine, and influenza vaccine in the past year among survey respondents from a convenience sample at the Minnesota State and County Fairs (August 2023).

	Primary Reason for Never Receiving COVID-19 Vaccine N = 97	Primary Reason for Not Receiving COVID-19 Bivalent Booster N = 382	Primary Reason for Never Receiving Influenza Vaccine N = 95	Primary Reason for not Receiving Influenza Vaccine Last Year N = 253
	No. (%)	No. (%)	No. (%)	No. (%)
I’m concerned about potential side effects	35 (36.0%)	61 (16.0%)	7 (7.4%)	19 (7.5%)
I don’t think the vaccine is effective	26 (26.9%)	24 (6.3%)	24 (25.3%)	41 (16.2%)
I am not worried about getting sick	9 (9.3%)	79 (20.7%)	26 (27.3%)	65 (25.7%)
Vaccines are against my religious or personal beliefs	7 (7.2%)	1 (0.3%)	5 (5.3%)	4 (1.6%)
I did not know about this vaccine	3 (3.1%)	51 (13.4%)	1 (1.1%)	1 (0.4%)
I have a medical condition and cannot get vaccinated	2 (2.1%)	3 (0.8%)	4 (4.2%)	3 (1.2%)
I did not know where to get one or could not find one	1 (1.0%)	11 (2.9%)	2 (2.1%)	6 (2.4%)
Getting vaccinated is too time consuming or expensive	-	11 (2.9%)	-	13 (5.1%)
Other	12 (12.3%)	104 (27.2%)	15 (15.8%)	66 (26.1%)
I don’t know	-	29 (7.6%)	3 (3.2%)	29 (11.5%)
Prefer not to answer	4 (4.1%)	8 (2.1%)	8 (8.4%)	6 (2.4%)

**Table 3:** Unadjusted and adjusted odds ratios and 95% confidence intervals from multinomial logistic regression for combined intention to receive routine COVID-19 and influenza vaccine in the coming year among respondents from convenience sample at 2023 Minnesota State and County Fairs (N = 867).

	Unadjusted						Adjusted *					
	Neither Vaccine (vs. Ref Both Vaccines)		Influenza Vaccine (vs. Ref Both Vaccines)		COVID-19 Vaccine (vs. Ref Both Vaccines)		Neither Vaccine (vs. Ref Both Vaccines)		Influenza Vaccine (vs. Ref Both Vaccines)		COVID-19 Vaccine (vs. Ref Both Vaccines)	
	OR	95% CI	OR	95% CI	OR	95% CI	OR	95% CI	OR	95% CI	OR	95% CI
Politics (ref: Democrat)												
Republican	10.3	(5.2–20.3)	7.9	(4.0–15.6)	1.8	(0.7–1–4.8)	12.8	(6.2–26.6)	8.7	(4.2–17.9)	1.9	(0.72–5.3)
Independent	3.9	(2.0–7.6)	2.9	(1.5–5.6)	0.92	(0.40–2.1)	4.3	(2.1–8.7)	3.2	(1.6–6.4)	0.95	(0.40–2.3)
Other	5.2	(2.2–12.0)	3.8	(1.6–9.0)	1.0	(0.29–3.5)	3.2	(1.3–7.6)	3.0	(1.2–7.2)	0.72	(0.20–2.6)
Strong belief vaccines are collective action (ref: no)	0.06	(0.04–0.10)	0.14	(0.08–0.25)	0.74	(0.32–1.7)	0.06	(0.03–0.11)	0.13	(0.07–0.24)	0.64	(0.25–1.6)
COVID-19 vaccine requirement (ref: no)	0.66	(0.29–1.52)	0.77	(0.37–1.58)	2.5	(1.3–5.0)	0.42	(0.16–1.12)	0.73	(0.33–1.6)	2.0	(0.94–4.4)
Influenza vaccine requirement (ref: no)	0.19	(0.07–0.54)	2.0	(1.1–4.0)	0.19	(0.06–0.57)	0.18	(0.06–0.59)	1.8	(0.88–3.7)	0.16	(0.05–0.58)

* Adjusted for age, gender, annual household income, educational attainment, and medical condition risk of severe illness.

## Data Availability

The dataset is available on request from the authors.
